# Application of lab-on-a-chip multiplex cassette PCR for the detection of enterohemorrhagic *Escherichia coli*

**DOI:** 10.1186/s12866-019-1463-1

**Published:** 2019-05-14

**Authors:** Dammika P. Manage, Jana Lauzon, Lynn M. McMullen, Linda M. Pilarski

**Affiliations:** 1 Department of Agricultural, Food and Nutritional Science, University of Alberta, Edmonton, AB T6G 2P5 Canada; 2 Department of Oncology, University of Alberta and Cross Cancer Institute, 11560 University Ave, Edmonton, AB T6G 1Z2 Canada

**Keywords:** Rapid detection, Lab-on-a-chip, PCR, Food pathogens

## Abstract

**Background:**

Fast molecular detection methods benefit from ready-to-run lab-on-a-chip molecular assays with minimum preparation time. Detection efficiency of such methods can improve if multiple targets are detected simultaneously per given reaction. Detection of food pathogens, i.e. *Escherichia coli* (*E. coli*), is generally performed in two stages with the detection of multiple targets in each stage.With simultaneous testing, screening for pathogens is fast and efficient.

**Results:**

In this study, we show the application of multiplex PCR performed on a ready-made cassette to detect 10 targets each for eight samples known to harbor *E. coli*. In cassette PCR, the aluminum cassette (38.6 mm × 31.4 mm) contains 10 trenches having a total of 50 capillaries with microliter volumes of desiccated acrylamide gels holding all reagents required for the PCR including internal positive and negative controls. The gel contains LCGreen dye to detect double stranded DNA. Fluorescence monitoring allows the detection of the amplified products by melt curve analysis. In this application, each of the five capillaries in a given trench contains two of the primer sets for the detection of 10 targets in pathogenic *E. coli*, namely, O157, Eae, Stx1, Stx2 and six O-antigen genes. Primer specificity was confirmed. Each trench tests one sample. Eight minimally processed enriched beef carcass swab samples were analyzed for parallel detection of 10 targets within 1 h and 15 min. Samples were delivered to the capillaries by capillary forces thereby hydrating the gels. Multiplex cassette PCR results were confirmed with conventional multiplex PCRs performed in a commercial real-time PCR system.

**Conclusions:**

Cassette PCR technology is ideally suited to multi-target detection of pathogens in food products. The cassette performs multiple PCR reactions in parallel, with multiplex detection of targets within each reaction unit. Cassette PCR/ melt curve analysis results for the simultaneous detection of 10 targets of pathogenic *E.coli* in beef carcass swab samples were confirmed with a conventional real-time PCR/ melt curve analysis as well as with agarose gel electrophoresis. Although designed for the detection of *E. coli*, this multiplex cassette PCR technique can be applied to any other assay where the fast detection of multiple targets is required.

## Background

In multiplex real-time PCR, multiple primer sets are amplified in a single reaction unit targeting different genes and producing multiple amplicons preferably having different sizes. Amplifying multiple sequences together and hence performing the entire test set simultaneously is time and cost efficient. Examples where multiplex PCRs can be used are the detection of multiple pathogens or multiple serogroups of one pathogen [1–6], series of cancer markers [7, 8], or series of sexually transmitted diseases [9, 10] in a given sample.

Multiplex real-time PCRs are mostly performed with many primers coupled with probes that are labeled with different colored fluorophores [11]. The detection of such PCR products requires a sophisticated instrument with a wide spectrum light source to excite each fluorophore as well as multiple light filters to measure the fluorescence emission from each dye. Achieving a real-time PCR curve or a melt peak for a given primer set with a given color confirms the presence of the targeted DNA in the tested sample. Probes labeled with different colored fluorophores as well as the instruments capable of detecting multiple colors are costly. Another option would be to do multiplex real-time regular PCRs with primers that amplify products of different sizes that can be resolved with no need for probes, by adding an intercalating fluorescent dye to the reaction, and using the position of the melt peaks to detect amplification of multiple DNA targets [12, 13]. An instrument using intercalating dyes needing to detect only one color is relatively cost effective. However with a single color, since the position of the melt peak of the amplicon is used for the diagnosis, primers must be designed to produce PCR products with distinct melt peaks.

Here we adapted our cassette PCR technology to perform multiplex PCR combinations. Cassette PCR is performed in a sub-microliter volume of gel media (~ 6 μL) inside a 6 mm long glass capillary [14–18]. The cassette can contain a large number of capillaries that are capable of performing individual PCRs simultaneously. The gel inside the capillary is desiccated containing all the PCR reagents, including an intercalating dye. The user need only add the sample. The desiccated noodle shaped gel inside the capillary allows the sample delivery by capillary forces. Capillary reaction units made with different primers are placed inside wax laid trenches in the cassette. Sample is subsequently administered to all of the capillaries in a given trench by delivering it to the capillary unit at either end. This enables the sample to flow through all the capillaries in the trench thereby hydrating all aligned gels with a given sample. Pre-assembled cassettes can be stored in airtight sealed bags in the refrigerator for prolonged period of time until the time of use. [17]. We have extensively tested our cassettes for detection of a single target per a given capillary for sexually transmitted diseases in genital swabs or in urine [16], cancer markers in buccal swabs [15], and for detection of viruses in whole blood, although with a different gel geometry [19]. The cassette design and the capillary layout are flexible for adapting to the required application. Our latest cassette geometry comprises of 50 capillaries laid in 10 trenches, with 5 capillaries per trench [18]. It can test up to 8 samples for 5 targets each (with singleplex PCR), including integrated positive and negative controls. Positive control capillaries carry target DNA embedded in the gel, which is hydrated by water instead of sample. The cassette is made of aluminum for faster heat exchange. Our second generation PCR instrument, the “GelCycler MarkII”, has sensitivity to detect 1–3 DNA targets per capillary [18]. It contains heated blocks maintained at different temperatures with the cassette automatically moved for each step of the thermocycling and melt curve analysis. The detection is performed by acquiring the images of the entire cassette with the fluorescence emitted by LCGreen, a non-saturating stable intercalating dye that binds to double stranded DNA [20].

In this manuscript, we demonstrate the ability of cassette PCR to carry out multiplex detection of markers for enterohemorrhagic *Escherichia coli* in enriched beef carcass swab samples. Multiplex PCRs were performed simultaneously on a GelCycler MarkII for 10 targets in 5 capillary units for detecting four Shiga toxin-producing *E. coli* (STEC) O157 markers and six most frequent non-O157 serogroups namely, O26, O45, O103, O111, O121, and O145. The validity of our results was confirmed with a conventional real-time PCR performed on a commercial instrument with PowerUp SYBR Green master mix (Thermo Fisher Sci.) and with conventional agarose gel electrophoresis.

STEC infections frequently cause bloody diarrhea and hemolytic uremic syndrome (HUS), a severe complication characterized by renal failure that can be fatal. Approximately 265,000 illnesses and 3600 hospitalizations occur due to STEC infections annually in United States alone [21] raising major concerns for food safety. Current commercial detection methods for detection of STEC in North America are performed in two stages where the initial PCR screening is performed for the intimin *Eae* gene, shiga-toxin genes (*stx*1 and *stx*2) and O157:H7 [22]. If this screening scores positive for *Eae* and shiga toxin genes, a confirmation assay is performed targeting non-O157 six O-antigen specific genes. Otherwise, no further testing is performed. However, after the *E. coli* outbreak in Fenugreek sprouts in Germany in 2011 affecting 3950 people and causing 53 deaths, the EU has made O-antigen testing mandatory [23]. The tests should be negative for STEC, O157 as well as O-antigen serogroups and *Salmonella* species in order for the sprouts to be released to the market. In such cases, performing the entire *E. coli* test for STEC as well as O-antigen testing in one test device at once would be an advantage.

Although we chose food safety application targeting STEC markers to demonstrate the multiplex cassette PCRs, this technique can be applied to any other assay that requires the detection of many targets.

## Results

**Fig. 1:**
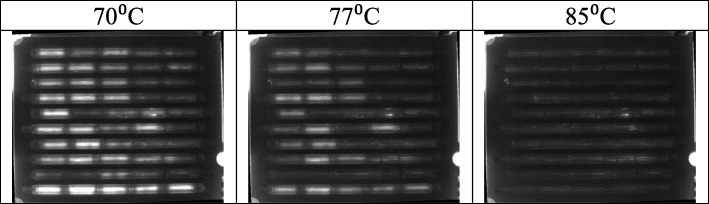
CCD images of the cassette during the MCA at three representative temperatures

The LCGreen dye used in the PCR is highly fluorescent when intercalated with double stranded DNA. As the PCR progresses and more amplicons are produced, the fluorescence signal in the capillaries increases. During the melt curve analysis (MCA), amplicons melt into single stranded DNA at their melting temperature causing the dye to fall off resulting in a sharp loss of fluorescence. The negative derivative of this fluorescence vs. the temperature shows a peak at a specific temperature for a given amplicon amplified by a given primer set. The presence of a peak at the correct melt temperature allows the identification of each target. The CCD images of the cassette at 70 °C, 77 °C and 85 °C during the MCA are shown in Fig. 1. The entire cassette contained 50 capillaries covering 10 primer sets with each column having two multiplexed primer sets. Eight enriched and minimally processed beef carcass swab samples were amplified by PCR and the products were melted in the first 8 trenches while the negative and positive controls were amplified and melted in the 9th and 10th trenches respectively. A summary of the genotyping results for singleplex PCRs performed on each beef carcass swab sample with cassette PCR as well as with conventional PCRs for each of the 10 primer sets (targeting four STEC markers and six O-antigens) are shown in Table 1. This defines the pathogen profile for each sample that was evaluated using the multiplex strategy. Note that some samples appear to contain more than one *E. coli* strain, based on the O antigens detected, as might be expected from samples taken in the field.

**Table 1:** Genotypes of beef carcass swab samples 1–8 confirmed by single-plex PCR

	single-plex PCR confirmation									
Sample #	Eae	Stx1	O157	Stx2	O26	O45	O103	O121	O111	O145
1	+	+	–	+	+	–	+	–	–	–
2	+	+	–	+	–	+	–	–	–	+
3	+	+	+	+	+	+	+	–	–	–
4	–	+	–	+	–	+	–	–	–	–
5	+	+	–	–	–	–	+	–	–	–
6	+	–	–	+	–	–	–	+	–	–
7	+	+	+	+	–	–	–	–	–	–
8	+	–	–	+	–	+	+	–	–	–

**Fig. 2:**
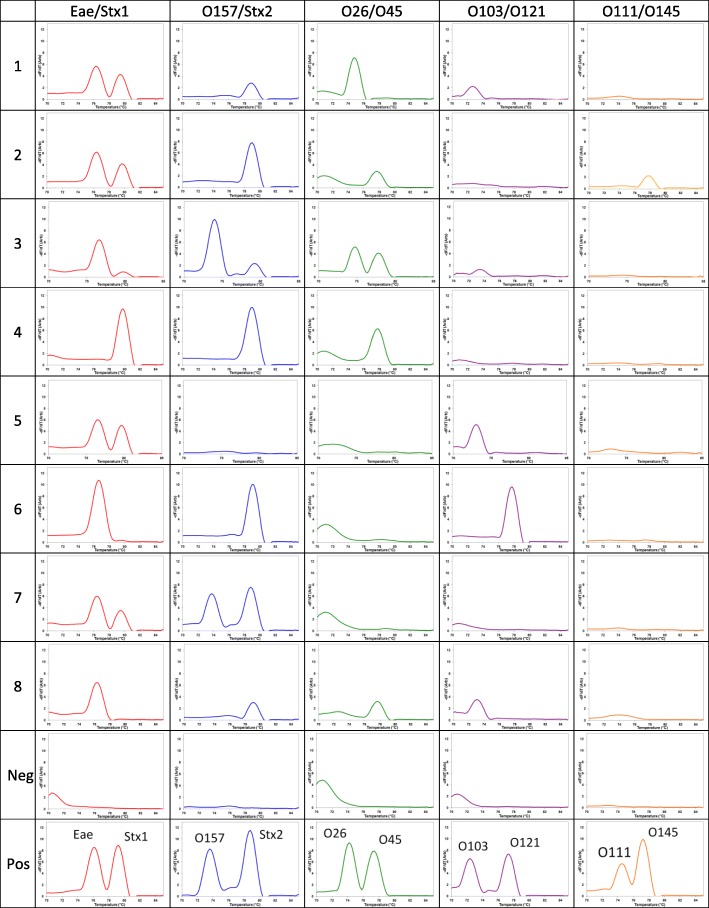
MCA curves of the fifty capillaries in the entire cassette. First eight rows show eight beef carcass swab samples while the 9th and 10th rows show the negative and positive controls respectively. Each of the rows corresponds to a trench in the cassette. Each column represents a set of capillaries with a given multiplex primer-set. No cross contamination between capillary reaction units was ever detected

The melt peaks for the entire cassette are shown Fig. 2. Each plot represents one capillary containing two primer pairs per given sample. Each row represents a trench in the cassette with the five multiplex capillaries containing the two primer-pairs stated in the column titles. Upon the heating of the cassette during the MCA, the disappearance of the fluorescence in each capillary in Fig. 1 corresponds to a melt peak in the Fig. 2. If the target DNA is present in the sample for one of the two primer pairs in a given capillary, that DNA is amplified hence showing a single melt peak at the corresponding temperature. If the target DNA is present for both primer pairs in a given capillary, both targets are amplified showing two melt peaks. The presence or the absence of a melt peak at a given temperature was used to determine whether the sample is positive or negative for each target. Individual melt peak positions for four STEC markers and six non-O157 O-antigen markers were determined by performing singleplex cassette PCRs with positive controls and the specificity of each primer was determined by performing singleplex PCRs using the O-antigen positive controls (data not shown). Combination of the primer sets were chosen such that the melt peaks are distinct from each other for easy identification. Since, each plot in Fig. 2 represents an individual capillary containing two primer pairs, the presence or absence of a peak in Fig. 2, and hence the pathogen profile for each sample, can directly be compared with Table 1. The multiplex derived genotype profiles for each sample match those expected from Table 1, based on the melt peaks shown in Fig. 2. As seen in Table 1, some beef carcass swab samples are positive for multiple O-antigens, ie. sample#3 positive for O26, O45 and O103. When 308 retail ground beef samples were tested for *E. coli* O157:H7, 6 non-O157 STEC serogroups by Wasilenko et al., it was found that some samples carried up to 3 O-antigens as well [24].

**Fig. 3:**
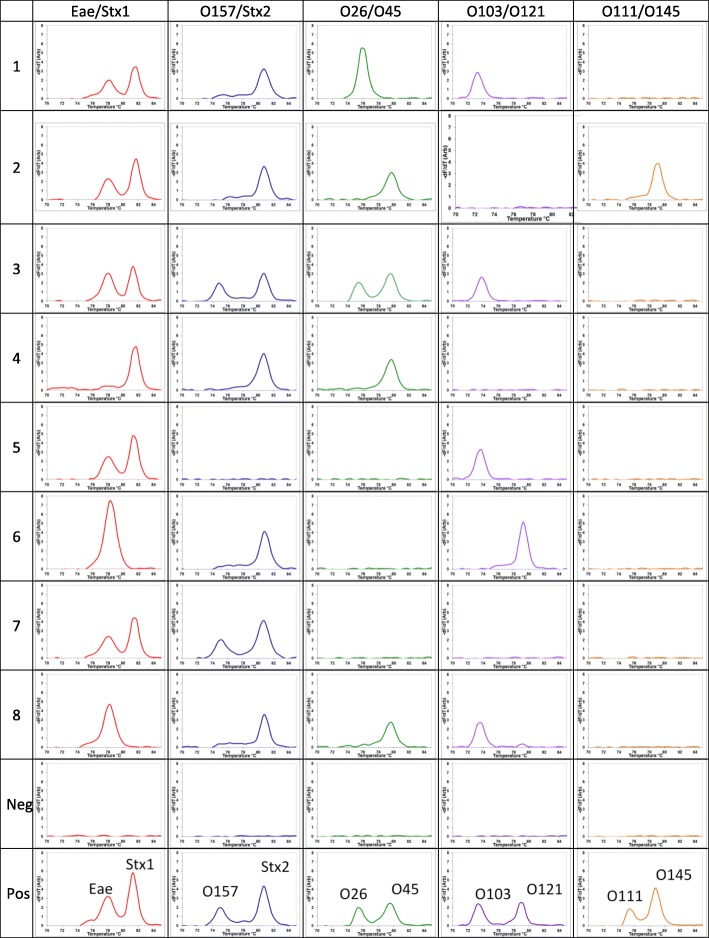
MCA data from conventional real-time PCR machine on the same samples run on the cassette PCR in Fig. 2

Melt peaks from the real-time multiplex PCR run on the commercial instrument (Stratagene Mx4000) with the ready-made PowerUp SYBR Green master mix are shown in Fig. 3. Each plot in Fig. 3 represents a PCR reaction performed in a single tube with two primer pairs. If the sample contains only the target DNA for a single primer pair, only one melt peak is present in the given PCR reaction tube. If the sample contains target DNAs for both primer pairs, two melt peaks are present in the given PCR reaction tube. Each row in Fig. 3 represents a sample or a PCR control. First 8 rows show 8 enriched beef carcass swab samples while the last two rows show the negative and positive controls respectively for the five multiplex reactions run in five separate tubes per each row. The layout of Fig. 3 can be directly compared with the layout of Fig. 2.

**Fig. 4:**
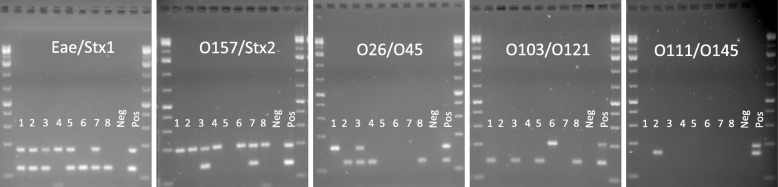
Amplified products of five multiplex PCR reactions from the conventional real-time PCR run on 2% agarose gel. The marker used in each side of each gel is the GeneRuler 1 kb Plus DNA ladder with the last three bands of 75, 200 and 300 bp. Each gel represents a column in Fig. 3. The sample lanes from left to right in each gel are the beef carcass swab samples 1–8, negative and positive controls as seen in Fig. 3

Figure 4 shows the agarose gel images for the amplicons of each reaction performed in the real-time conventional PCRs (shown in Fig. 3). Each of the five images represents two primer pairs for the 8 beef carcass swab samples and the negative and positive controls and indicates the size of each product amplified by the primers shown in Table 2, providing further confirmation of the test accuracy and of primer specificity.

**Table 2:** Primer sequences for *E. coli* (STEC) and six O-antigen amplifications

Primer	Sequence	Length (bp)	Reference
O157-F	TCGTGACAACCATTCCACCTT	123	This work
O157-R	GCGCTGAAGCCTTTGGTTCT		
Eae-F	CATTGATCAGGATTTTTCTGGTGATA	102	[29, 30]
Eae-R	CTCATGCGGAAATAGCCGTTA		
Stx1-F	GTGGCAAGAGCGATGTTACGGTTTG	182	[31]
Stx1-R	ATGATAGTCAGGCAGGACGCTACTC		
Stx2-F	ACGAGGGCTTGATGTCTATCAGGCG	200	[31]
Stx2-R	GCGACACGTTGCAGAGTGGTATAAC		
O26-F	GTATCGCTGAAATTAGAAGCGC	158	[30, 32]
O26-R	AGTTGAAACACCCGTAATGGC		
O45-F	GGGCTGTCCAGACAGTTCAT	101	[5]
O45-R	TTGAGACGAGCCTGGCTTTGATAC		[3]
O103-F	TATCCTTCATAGCCTGTTGTT	133	[6]
O103R	ATATTAAGAGGAAGAGCGCATAGA		This work
O111-F	GGAATAATCGACCGGCCAAA	199	This work
O111-R	TAATGTGTTGCCTCGCCTTC		
O121-F	TGTTGGCTAGTGGCATTCTGA	212	This work
O121-R	TTCTGCATCACCAGTCCAGA		[5]
O145-F	ACGTGAAAAAGCCTCGTAGTG	162	This work
O145-R	TTGGTGGTACTGTGTCCGC		

## Discussion

The MCA results from the multiplex cassette PCR shown in Fig. 2 are fully concordant with the conventional real-time multiplex PCR and MCA data with PowerUp SYBR Green master mix, as shown in Table 1. This confirms the feasibility and accuracy of cassette multiplex PCR for molecular diagnostics where there is a demand for the detection of multiple targets. The peak strengths in each multiplex reaction in the two methods vary since the primer concentration ratios are not exactly the same. In addition, some peaks in Fig. 3 are stronger than those in the Fig. 2, most probably due to the increased amplification due to five additional PCR cycles performed in the conventional method. However, all the peaks from both methods are in agreement. The melt peak temperatures for amplicons of the same genotype in the two test systems differ somewhat, likely due to the difference salt concentrations in the two PCR reagent cocktails. As long as the melt peaks of the samples are compared to the positions of the positive controls in the same systems, the method is accurate. The sizes of gel bands in Fig. 4 also confirm the amplification of the correct product in each multiplex reaction carried out with the primers specified in Table 2.

Recent studies suggest that there are viable but non-culturable (VBNC) [25, 26] and also dead cells [27] in addition to the viable cells of pathogenic bacteria. These VBNC or dead cells can produce false positives in PCR. However, with the dilution factors involved during the enrichment of beef carcass swabs as well as during the sample processing for our PCR, it is highly unlikely that we detect VBNC cells or dead cells in our capillary PCR units.

Although used here for the simultaneous detection of pathogenic *E. coli* and the O-antigens, cassette multiplex PCR can be applied to any application where the amplification of many targets is required. The cassette is pre-made and sealed with capillaries containing the gels with all the reagents required for PCR except the sample. The user needs only to open the sealed plastic encasing, load the samples to the sample trenches and add water to the negative and positive control trenches. Since handling of conventional positive controls, used in higher concentrations, sometimes cause contamination issues, having an integrated positive control is an advantage. In our cassette, the sample delivery is performed simply via the capillary forces; hence there is no need for applying pressure that often involves pumps and valves. When handling microliter volumes in many lab-on-chip devices, evaporation of the sample and the reaction components is a problem during the experiment especially when heating is involved. Evaporation in such devices is prevented by applying seals with pressure that again involves extra components in the instrument sometimes requiring an external vacuum line or a pump. The wax in our PCR cassette prevents the evaporation by sealing the ends of the capillaries at the start of the PCR. The liquid wax also segregates the individual capillaries (reaction units) avoiding any possibility of contamination of the primers or the amplicons among the adjacent capillaries. The absence of any cross contamination in cassettes has been experimentally confirmed numerous times [14–17]. The aluminum cassette is thermally highly conductive, allowing faster PCR cycles. The physical barrier presented by the aluminum walls of the trenches prevents any light bleed between the adjacent trenches and also provides more efficient heat transfer to the capillaries. The multiplex PCR data shown in Fig. 2 from the cassette was obtained within 1 h 15 min compared to the data shown in Fig. 3 from the conventional multiplex real-time PCR machine that was obtained within 3 h. The data from the cassette PCR is comparable to the data obtained from the conventional real-time PCR data. This confirms the capability of our cassette PCR of performing multiplex PCR for detecting multiple targets simultaneously at low cost and in significant faster time.

Methods involving multiplex PCRs for typing STEC in liquid media have been published with different target combinations. Anklam et al. reported four real-time PCR assays covering all STEC and six o-antigen targets [1]. In each assay, different colored fluorescence probes were used with targeted primer combinations and purified DNA from bacterial strains was used to verify the assays. O157 and six O-antigens were detected by Paddock et al. with a single multiplex conventional PCR assay where primers produced seven targets with different lengths for size identification by agarose gel electrophoresis [5]. BAX, a commercial system, uses four multiplex PCR assays to cover all STEC and O-antigen targets where real-time PCR is performed followed by MCA [28]. These BAX assays come with all the PCR reagents in each tube where the user needs to add the processed sample. All the real-time multiplex methods involve adding the sample to many tubes containing different assays to cover all the STEC and O-antigen targets. To our knowledge, this is the first ready-made cassette application where the multiplex PCR is performed in a gel media followed by MCA for detecting all STEC and O-antigen targets. The sample is added to all five assays covering 10 targets at once by capillary forces.

## Conclusions

We demonstrated that our ready-made gel cassette can perform multiplex PCR hence increasing the throughput of the detection. The cassette contains capillaries with desiccated gels comprising of all the reagents required for PCR for multiple primers and the LCGreen dye for monitoring the amplification as well as for detection of the amplified products by MCA, including internal positive and negative controls. The cassette geometry has the flexibility to change depending on the application. It can also be stored at a refrigerated temperature for prolonged periods of time, until the time of use. The current multiplex PCR cassette can detect up to 10 targets of 8 samples with respective negative and positive controls. As an application of this technology, we analyzed eight enriched beef carcass swab samples each for 10 targets in pathogenic *E. coli*. Since the detection is performed by taking CCD images of the entire cassette at each PCR cycle and at each temperature in MCA, both PCR/MCA takes only 1 h and 15 min to complete. Cassette PCR results were confirmed with a conventional real-time PCR/MCA and with agarose gel electrophoresis.

## Methods

### Capillary preparation

**Fig. 5:**
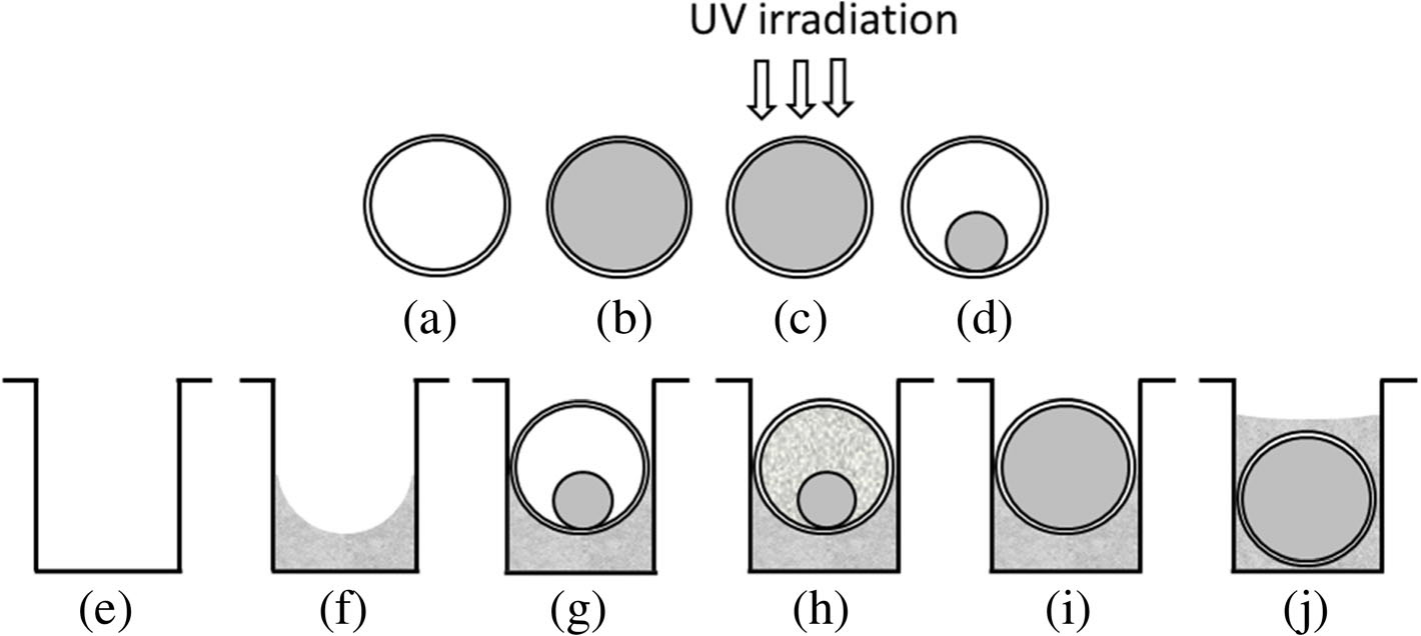
Preparation of the PCR cassette (width 31.4 mm, length 38.6 mm, and height 3.1 mm): (**a**-**d**) capillary preparation and (**e**-**j**) cassette preparation (only one trench of the cassette is shown for the simplicity): (**a**) empty glass capillary (length 6 mm, diameter 1.6 mm), (**b**) capillary is filled with the gel/PCR reaction mix, (**c**) capillary is exposed to UV to polymerize the gel and (**d**) polymerized gel is desiccated inside the capillary, (**e**) empty trench (length 30.5 mm, width 1.8 mm, depth 2.4 mm), (**f**) 40 μL of molten wax is poured and allowed to solidify, (**g**) capillary with the desiccated gel is placed on the wax in the trench (total of 5 capillaries were placed per trench, capillaries touch each other), (**h**) sample is pipetted by administering it to the last capillary in the trench and letting all five capillaries fill by capillary forces, (**i**) the desiccated gel is hydrated by the sample, and (**j**) at the start of the PCR, wax melts causing the capillary to sink to the bottom of the trench and segregating each capillary as an individual reaction vessel.

The capillaries used to do multiplex PCRs were 1.6 mm in diameter and 6 mm in length as explained earlier [14]. The sequences of the primers used for multiplex detection of shiga toxin-producing *E. coli* and O-antigens are shown in Table 2. Capillary as well as the cassette preparation steps are shown in Fig. 5 and are described below.

For the multiplex PCR, 100 μL gel-PCR reaction mix consisted of 20 μL of 5xPCR buffer [333 mmol/L tris sulfate, pH 8.6, 83 mmol/L (NH4)_2_SO_4_ (Sigma, St. Louis, MO) and 40% sucrose (Sigma)], 30 μL of 40% trehalose (Acros, New Jersey, USA), 2 μL of 100 mmol/L MgCl_2_ (Ambion, USA), 2 μL of 10 mmol/L dNTP (Thermo Fisher Sci.), 2 μL of 2% bovine serum albumin (Ambion), 10 μL of 10x LCGreen Plus (BioFire, Utah, USA), 6 μL of 20 U/μL Taq polymerase, 10 μL of a 40% acrylamide (Fisher) + 4% bis-acrylamide aqueous solution (N,N-methylene bisacrylamide, Bio-Rad, Hercules, CA), 2 μL of 3% azobis (Wako Bioproducts, Richmond, VA), 1 μL of 10% N,N,N′,N′ tetramethylethylenediamine (Sigma), primers (concentrations are shown below) and water. The mixes were vortexed, centrifuged, and loaded into the capillaries. Polymerization and the desiccation of the gel/PCR mix were completed as shown in Fig. 5a-d. Detailed description of this process was previously published [14, 15].

The five multiplex reactions consisted of primers: Eae(1 μM) /Stx1(0.3 μM), O157(1 μM) /Stx2(0.3 μM), O26(0.6 μM)/O45(0.3 μM), O103(1 μM)/O121(0.3 μM), and O111(0.7 μM) /O145(0.4 μM) with the final primer concentrations shown in brackets.

For the positive control multiplex capillaries where the template was embedded into the gel, the templates were added to the gel/reaction mix before loading the capillary and desiccating the gel. Each of these reaction mixes was similar to the above but the template or templates were added replacing the volume of the water. For the Eae/Stx1 and O157/Stx2, 4 μL of 1:10 diluted heat killed STEC culture was added while for O-antigen multiplex mixes, 4 μL of each of the 1:10 diluted heat killed O-antigen culture were added. Information of the templates is given in the sample section.

### Preparation of the cassettes

**Fig. 6:**
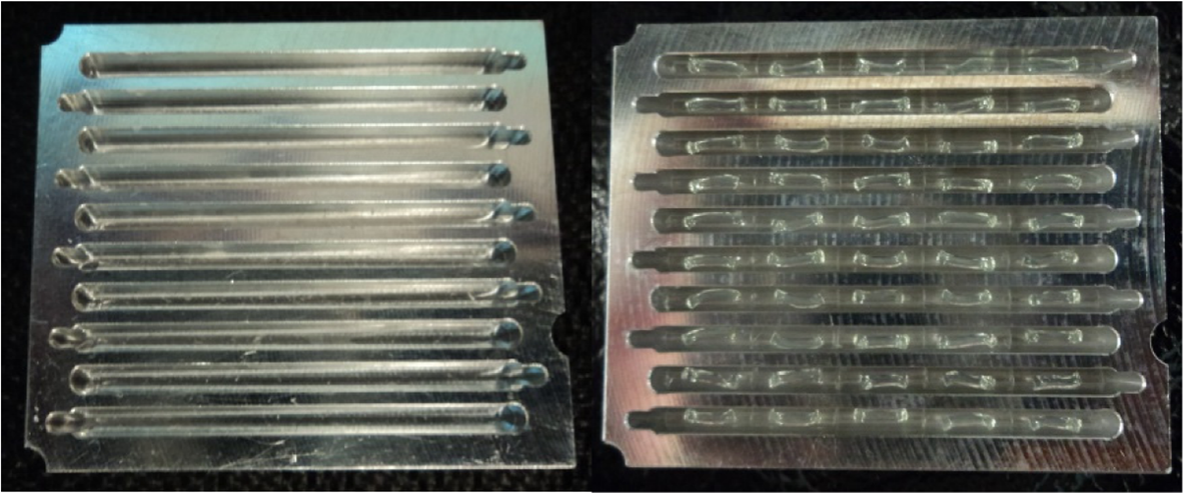
An empty cassette (left), and a cassette loaded with 50 capillaries (right). Cassette is 38.6 mm × 31.4 mm × 3.1 mm in size

The aluminum cassette with 10 trenches was used for this work [18]. The steps for making the cassette are shown in Fig. 5e-j. Briefly, the cassette was heated to ~ 70 °C and each of the 10 trenches was filled with ~ 40 μL of molten wax (Surgipath Paraplast X-tra, Leica Microsystems, Deerfield, IL). It was cooled to room temperature and 5 capillaries with all the gel/reagents desiccated inside that required for multiplex PCR were laid in each of the first 9 trenches with the order of Eae/Stx1, O157/Stx2, O26/O45, O103/O121, and O111/O145 (Fig. 6). Positive control capillaries with DNA embedded inside were placed in the 10th trench in the same order.

### Samples

Pathogenic *E. coli* strains used as the positive controls are STEC-O157:H7 ATCC 43895, O26:H11, O45:H2, O121:H19, O103:H2, O111:NM, and O145:HN. A single colony of each of these strains was suspended in 10 mL TSB (Tryptic soy broth, Bacto, Le Pont de Claix, France*)* and incubated for 18 h at 37 °C. The final culture was heated at 90 °C for 10 min to inactivate the pathogens.

Enriched beef carcass swabs were used for actual sample testing. Aliquots of enriched media of beef carcass swabs (collected from provincially-licensed abattoirs in Alberta) that had been enriched at 42 °C for 18.5–23 h in mTSB (modified tryptic soy broth) with 1:6 ratio, were received from Alberta Agriculture and Forestry. Eight μLs of this overnight culture was mixed with 82 μL of proprietary lysis buffer (Amplicet Inc., Edmonton AB, Canada, patent pending). The mix was vortexed and heated at 55 °C for 15 min followed by 97 °C for 4 min. This proprietary lysis buffer in conjunction with the heat treatment removes the PCR inhibitors. Therefore, this lysed sample was directly added to the capillaries or to the commercial PCR mix.

### Sample loading to the cassette

In order to hydrate the gel/PCR reaction mix, 35 μL of sample was loaded to the 5 capillaries in each trench by administering the sample to the capillary at one of the ends [14] (Fig. 5h), as described above. The sample hydrates the desiccated gel/reaction mix within 10 min (Fig. 5i).

For the trenches containing positive and negative control capillaries, water was added instead of the sample.

### GelCycler MarkII

**Fig. 7:**
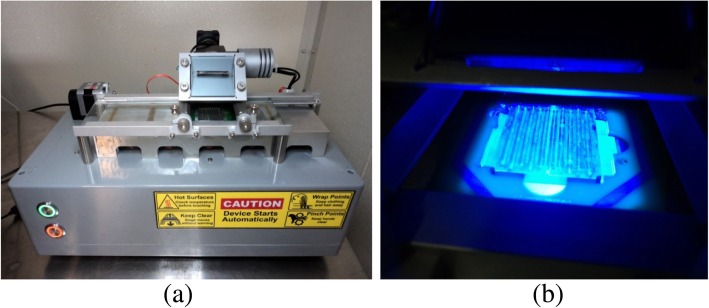
Photographs of (**a**) GelCycler MarkII (length 40 cm, width 25 cm, height 23 cm) and (**b**) illuminated cassette during the image acquisition

A photograph of the GelCycler MarkII thermocycler used for the cassette PCR is shown in Fig. 7a. The detailed description of the instrument was published earlier [18]. Briefly, it consists of 4 heater blocks. The far right block is an unheated loading block. From right to left (1 to 4), each heater block is maintained closer to annealing, extension, pre-denaturation, and denaturation temperatures that are programed to vary slightly depending on the cassette movement.

In a regular PCR cycle, the cassette moves from the denature block (4th) to the annealing block (1st) over the pre-denature block (3rd) and extension block (2nd). Once the anneal step is completed, it moves to the extension block. The camera for taking CCD images of the entire cassette is located above the extension block. At the end of the extension step in each PCR cycle, a CCD image is taken as seen in Fig. 7b. The cassette then moves to the pre-denature block that is kept slightly higher than denature temperature for few seconds to quickly raise the cassette temperature to close to the denature temperature. Then the cassette moves to the denature block and stays for the duration that is required for the denaturation step of the PCR. During this time, pre-denature block temperature is slightly lower than the denature temperature to receive the hot cassette and begin to cool it while the cassette is passing over the block. Each of the blocks except the denature block is programmed to change the temperature slightly to raise or cool down or/and maintain the required cassette temperature during each step of the PCR cycle [18].

Upon completion of the PCR, the cassette remains on the extension block where the temperature is raised from a user specified start temperature for performing MCA. The CCD images are taken with 0.2 °C intervals. MCA stops at a user specified temperature and the cassette moves to the loading block to complete the experiment.

### Conventional real-time PCR

For the conventional real time multiplex PCR, a Stratagene Mx4000 instrument was used in conjunction with the PowerUp SYBR Green master mix (Applied Biosystems). Each 10 μL master mix consists of 5 μL of 2x PowerUp SYBR Green master mix with primers Eae(0.6 μM)/Stx1(0.2 μM), O157(0.6 μM)/Stx2(0.2 μM), O26(0.6 μM)/O45(0.2 μM), O103(0.6 μM)/O121(0.2 μM), and O111(0.6 μM) /O145(0.2 μM), the final primer concentrations are shown in brackets. 3.4 μL of each processed beef carcass swab sample was added to the sample tubes while for the positive control tubes, 1.7 μL of each of the 1:10 diluted heat killed overnight cultures was added.

### Polymerase chain reaction and melt curve analysis (PCR/MCA)

For the GelCycler Mark II, DNA amplification was performed with a pre-denaturation step of 94 °C for 3 min, then 35 cycles of 94 °C for 11 s, 60 °C for 25 s, and 72 °C for 25 s, followed by a final amplification of 72 °C for 2 min. At each PCR cycle, a fluorescent image of the cassette is taken at the extension of the PCR cycle. Once the PCR starts, the wax melts and the capillaries sink to the bottom of the trench as shown in Fig. 5j. Upon the completion of the PCR, MCA was performed by heating the cassette from 70 °C to 85 °C and the CCD images were taken at 0.2 °C degree intervals. This instrument is controlled via a laptop computer through a LabVIEW interface.

For the Mx4000 real-time PCR instrument, PCR was performed according to the instructions for PowerUp SYBR Green master mix with 50 °C for 2 min, 95 °C for 2 min, 40 cycles of 95 °C for 15 s, 60 °C for 15 s, and 72 °C for 1 min. The MCA consisted of a dissociation step by heating to 95 °C and a cooling down step to 65 °C. Then the amplified products were heated up to 95 °C with 0.5 °C increments while collecting fluorescence intensities at each temperature. Upon completion of PCR and MCA, these samples were run on conventional agarose gel to get the size confirmation of the amplicons.

## Abbreviations

(*E. coli*): *Escherichia coli*
CCD: Charged Coupled Device
DNA: Deoxyribonucleic acid
EU: European Union
MCA: Melt Curve Analysis
PCR: Polymerase Chain Reaction
STEC: Shiga toxin-producing *E. coli*
